# Employees buying organic food intention: An extension of the theory of planned behavior

**DOI:** 10.3389/fpsyg.2022.1054166

**Published:** 2022-10-24

**Authors:** MengMeng Jiang, Qiong Wu

**Affiliations:** School of Economics, Wuhan Donghu University, Wuhan, China

**Keywords:** buying intention, actual buying behavior, the TPB model, descriptive norms, moral responsibility, environmental concerns

## Abstract

A gradual increase in population and urbanization has increased the demand for global resources, which ultimately burdens the depletion of resources and challenges environmental sustainability worldwide. In recent decades, nature sustainability has been the biggest challenge encountered by humankind. In addition, the changing lifestyle and consumption patterns have enormously played a key role. However, the consumption pattern from the employee’s perspective suffers from the lack of research. Therefore, grounded on the theory of planned behavior (TPB), this research explores the antecedents and consequences of employees’ buying intentions in the world’s emerging market (China). Data were collected using a web-based link shared *via* WeChat and Q.Q.; resultants into 451 valid responses and partial least square structural equation modeling (PLS-SEM) using SmartPLS 4 have been administered for the analysis. Besides the insignificant effect of descriptive norms on buying intention and environmental concerns on purchasing behavior, other factors significantly impact purchase intention and actual buying behavior. This research witnesses a significant mediating role of buying intention. This research suggests that practitioners (i.e., marketers, government, policymakers, and environmental focus companies) develop strategies for public advertisement and launch a general message and campaigns both in urban and rural areas to prevent environmental sustainability and increases awareness related to organic consumption.

## Introduction

Since the last decade, individuals have shown increasing interest in buying organic food; thus, in 2022 global organic food market accounted for $ 259.06 billion and is expected to grow by $437.36 billion by 2026 at 14% (Research and Market, 2022). Organic food is a food item with a strict cultivating process (Le-Anh and Nguyen-To, 2020). It comprises items free from chemicals such as antibodies, fertilizers, genetics, herbicides, and organisms (Rana and Paul, 2017). Additionally, Rana and Paul (2017) stated that several items had been used to refer to organic food, including fresh, local, natural, and pure. There are several reasons, including environmental and personal health consciousness, for increasing interest (Tandon et al., 2021). Although there is an increasing consumer purchasing of organic food, it has been argued that researchers have given limited attention to a different context, such as understanding the predictors in developing regions and emerging markets (Le-Anh and Nguyen-To, 2020; Tandon et al., 2021). Additionally, it has been posited that inconsistencies are evidenced in empirical studies on how individuals perceive organic food and what influences them to buy? (Feil et al., 2020).

Employees are a fundamental unit of organizations; they not only spend many hours but “consume a third of their calories” during office hours (Clohessy et al., 2019). There is a need to explore the factors of their eating behavior and their behavior toward sustainable consumption (Wanjek, 2005). In this context, it has been argued that limited attention is given to understanding the domain of food consumption from the employee perspective (Salciuviene et al., 2019). They work in large companies with central canteens, cafes, and bars. Hence, their buying intentions play a key role in environmental sustainability. Therefore, this research was conducted to explore the antecedents and consequences of organic food in the context of employees working in emerging markets (China). In particular, the scholars used the manufacturing sector because it is criticized as a leading contributor to environmental pollution and under pressure by different stakeholders, including employees (Roscoe et al., 2019).

China is an emerging country as a leader in organic agriculture (Ahmed et al., 2021) due to technological change and structural change for economic development (Zhou et al., 2021), with a steady focus on green innovation technology (Li, 2022). In addition, rapid socio-economic development is accompanied by the industrialization of agriculture, food production, and modernization. Increasing concerns about environmental and health problems coupled with the rise in living standards and awareness regarding the benefits increase the demand for organic food in China. According to Global Organic Trade (2021) report, the 2021 organic food market size is $4.8 billion, representing 8% of global demand, and exported $2.91 billion of organic food (Ma, 2022). Although, per capital expenditure of $3.4 indicates that organic food consumption is still a relative niche category, and the organic food industry is in a nascent stage (Wei et al., 2022). In addition, it has been postulated that sustainable consumption has dramatically increased in urban cities (Shao, 2019). Besides, it has been noted that organic food sales account for less than 1% of total food sales (Qi and Ploeger, 2021). Thus, Qi and Ploeger (2019) argued that organic food still occupies a limited market share in the country. In this context, it is essential to explore motivators influencing Chinese employee’s intentions and actual behavior in buying organic food.

The theory of planned behavior (TPB), suggested by Ajzen (1991), is the most widely used theory in the domain of individuals’ intentions and actual behavior (Rana and Paul, 2017; Liu et al., 2020; Wei et al., 2022). Prior studies have used the TPB in different (i.e., individual, employees, and consumer) pro-environmental behaviors, such as organic food (Ahmed et al., 2021), energy-saving (Ru et al., 2021), and electric vehicles (Shalender and Sharma, 2021). The TPB consists of three factors: attitude, subjective norms, and perceived behavior control. However, it has been proposed that with the inclusion of other factors such as environmental concerns, environmental awareness (Ahmed et al., 2021), and moral norms (Liu et al., 2020), the explanatory power of the TPB theory can be strengthened. Although the TPB has been gradually employed in behavioral studies, it has been criticized for its lack of consideration of moral influences on green consumption behavior (Chen, 2016; Liu et al., 2020). It has been observed that the inclusion of moral obligations into the TPB model increases the proportion of explained variation of intention by 1–9% (Chen, 2016). Likewise observed, 7.9% by Chaudhary and Bisai (2018) and 3.58% by Sadiq et al. (2021) increase in explained variation by adding other factors. In this way, we have added descriptive norms, moral responsibility, and environmental awareness as additional to the TPB factors.

This study addresses 2-fold questions: (1) whether the TPB could explain the employee’s organic food consumption intentions and actual behavior in the context of China; and (2) whether additional predictors, descriptive norms, environmental concerns, and moral responsibility could improve the explanatory of the TPB model. Following the research questions, this research 2-fold aims (1) to explore the antecedents and consequences of the intention to buy organic food and (2) to examine the mediating role of buying intention between the TPB and extended factors and actual buying behavior.

This study has several contributions. First, several studies have explored the consumer purchasing intention for organic food, such as (Scalco et al., 2017; Qi and Ploeger, 2019; Sadiq et al., 2021; Yeow and Loo, 2022). Rarely a few studies explore the employee perspective, such as (Cornford and Pupat, 2019). Hence, this research contributes to the scant literature on factors influencing employees to consume organic food. Second, this research extends the scope of TPB by adding descriptive norms, moral responsibility, and environmental concerns. In addition, several studies explore the direct effect of determinants on individual intention and buying behavior (Maichum et al., 2016; Liu et al., 2020; Le and Nguyen, 2022); this study will contribute to the mediating role of buying intention between the TPB, extended factors, and buying behavior. Third, this research helps managers and practitioners comprehend factors influencing employees toward consumption behavior.

## Literature review and hypotheses development

### Theoretical foundation

The theory of planned behavior (TPB) is an addition to the theory of reasoned action, which is essential by the former model’s precincts in handling the behaviors on which people have insufficient uncoerced control (Ajzen, 1991). Derived from the theory of reasoned action, an individual’s intent to perform an assigned behavior is a major element in the TPB. Intentions are presumed to apprehend the motivational elements that affect behavior; they are clues of how strongly people are ready to try, of how considerable effort they are planning to place to perform that behavior. Nevertheless, it should be noted that a behavioral intent could find meaning in behavior only if the desired behavior is under voluntary control, such as the individual deciding whether to perform the behavior or not. Granting that few behaviors could in fact, fulfill this criterion quite efficiently, the performance of the majority relies at least to some extent on such non-motivational elements as the accessibility of indispensable opportunities and resources (including finances, time, abilities, and collaboration with others; Ajzen and Madden, 1986). To the level that an individual has the essential resources, opportunities, and desires to perform the behavior, he/she should thrive (Ajzen and Madden, 1986). We used TPB theory because it is widely used and suggested to understand the intention and buying behavior in the context of organic food consumption (Rana and Paul, 2017; Liu et al., 2020; Wei et al., 2022).

### Individual actual buying behavior

Ajzen (1991) states that “behavior is an individual’s noticeable response in a specific situation concerning a given target.” It is also denoted that behavior is a product of perception of behavioral control and compatible intentions (Ajzen, 1991). TPB supports the growth of purchase behavior (Ajzen and Madden, 1986). TPB describes the effects of motivation on the behavior that lies with the individual and their control to offer a model for consumer behavior. A plethora of research contains a general assessment of primary motivation that reflects the employee’s buying behavior for organic food, which shows environmental issues, health problems, health attributes, norms, and attitudes are some of the reasons (Kapuge, 2016; Oroian et al., 2017; Darsono et al., 2019).

### Attitude

Positive customer attitudes concerning organic food are likely to show positive behavior and buying intentions (Honkanen et al., 2006). It is suggested that customers’ positive attitude toward buying organic food is their perception of organic food as a healthy option compared to conventional substitutes (Sultan et al., 2020). An abundance of past evidence shows a significant correlation between positive attitudes and organic food buying behavior (Nguyen et al., 2019; Ali et al., 2021). This evidence also indicates a similar connection between significant attitude and buying intentions (Scalco et al., 2017). Attitudes play a part in describing the organic products of buying behavior by considering attitudes as the buying behavior explanatory sign (Darsono et al., 2019). In assessing organic food buying behaviors, evidence exhibits an inconsistency between the consumers’ favorable attitudes and actual buying behavior (Sultan et al., 2020; Ali et al., 2021). Several consumers exhibit a positive attitude toward buying organic food goods, but a relatively small number of consumers show their actual buying behavior (Rana and Paul, 2017; Ajzen, 2018). In South Korea, customers showed a negative attitude toward intention and buying organic products due to the high prices (Suh et al., 2012). In addition, there is an increasing trend in demand for organic food due to a lack of supply. Consequently, the prices increased in 2019, with prices of organic fruits reaching 13.9% (Zboraj, 2021). In this context, we argued that attitude might negatively influence buying intention and buying behavior. In addition, there is still a gap between people’s attitudes and actions (Rana and Paul, 2017; Singh and Verma, 2017). This gap between the employees’ likely attitude (intentions) and actual buying behavior is exhibited in the following hypotheses:

*H1a:* Attitude has a negative effect on employees' intention to buy organic food.

*H1b:* Attitude has a negative effect on an employee's actual buying behavior.

### Subjective norms

The subjective norm, also called the social norm, is culture oriented. Specifically, China is an emblematic collectivist nation, and subjective norms act more effectively in collectivist communities (Minton et al., 2018). Civilization growth shows that subjective norms significantly affect buying behaviors (Wang et al., 2019). When people see others in their community inclined to buy organic food, they also show interest in such products. Injunctive and descriptive norms are further categories of Subjective norms, independently affecting the buying intention (Chatzisarantis and Biddle, 1998). Descriptive norms are the perceived behavior of most people by others who show exemplary behavior. Because of conjunction psychology, descriptive norms usually exhibit conformity behavior, and individuals likely exhibit behavior like others to resemble the groups (Xu et al., 2022). Evidence prevails that descriptive norm is positively linked with intentions and buying behavior (Salmivaara et al., 2021). Previously, a few studies tested and evidenced a significant correlation between subjective norms and buying behavior (Minton et al., 2018; Testa et al., 2019). In addition, several studies reported significant correlations between subjective norms and purchase intention toward organic food (Qi and Ploeger, 2019; Sultan et al., 2020; Sadiq et al., 2021). At the same time, the injunctive norm is a person’s identification of a particular behavior, considering that others accept that behavior. The injunctive norm motivates individuals to take healthy food and avoid unhealthy food (Bevelander et al., 2020). Organic food buying has more environmental benefits compared to non-organic products. Based on the above discussion and argument, we hypothesize:

*H2a:* Subjective norms positively affect employees' intention to buy organic food.

*H2b:* Subjective norms positively affect employees' actual buying behavior.

### Perceived behavioral control

Perceived behavior control is defined as an individual’s own perception regarding the available resources (i.e., money, time, effort, etc.). It refers to “an individual’s own judgment about their capabilities to engage in a particular behavior” (Al-Swidi et al., 2014). Previously, it has been argued that perceived behavior control is formed by perceived barriers (price, availability) and ability (income or financial resources) that effect green consumption behavior (Al-Swidi et al., 2014; Testa et al., 2019). Strong perceived behavioral control leads to a certain behavior that promotes the actual buying behavior (Mirani et al., 2021). When customers trust the sufficiency of their skills to buy a specific product and have fewer hurdles in the buying process, they possess high perceived behavioral control, and their intentions to buy organic products will enhance (Wang and Li, 2018; Sultan et al., 2020; Ahmed et al., 2021). Previously, many studies have identified the significant correlations between perceived behavior control and buying intention (Le-Anh and Nguyen-To, 2020; Sultan et al., 2020; Ali et al., 2021). In contrast, Khayyam et al. (2021) reported an insignificant effect of perceived behavioral control. Thus, based on the above argument, discussion, and inconsistent results, we hypothesize as:

*H3a:* Perceived behavior control positively affects employees' intention to buy organic food.

*H3b:* Perceived behavior control positively affects an employee's actual buying behavior.

### Descriptive norms

The descriptive norms predict the resource-saving intentions (Warner, 2021) and have an appositive impact on the consumers’ environment-friendly buying intentions. In this case, if most individuals buy organic products, others’ similar intentions increase, leading to enhanced behavioral intentions (Salmivaara et al., 2021). Most employees collectively buy organic products, which enhances their environmental buying consciousness. It is observed that descriptive norm has a stronger impact on buying intention compared to injunctive norms (Xu et al., 2022). Khan et al. (2022) suggested that descriptive norms were influential in developing normative and sustainable intentions. Literature also shows evidence that descriptive, injunctive, and social norms were individually examined to assess their influence on behavioral intentions, and all of them were positively related to behavioral intentions (Doran and Larsen, 2016). Being a predictor of TPB, the descriptive norm enhances the fluctuation in intentions. Thus, the confirmatory relationship between the descriptive norms and intentions shows the likely existence of predictive power of the variables, offering high encouragement for further studies (Ham et al., 2015; Gao et al., 2017; Qalati et al., 2022). This study proposes the following relationships:

*H4a:* Descriptive norms positively affect employees' intention to buy organic food.

*H4b:* Descriptive norms positively affect employees' actual buying behavior.

### Moral responsibility

Several studies have been dedicated to enhancing the explanatory power of TPB in predicting behavior. Some scholars have joined the TPB with other theories, and some have added more elements to explain it in more detail and in different aspects. Ajzen (1991) states that moral obligation (responsibility) could enhance the prediction force of TPB concerning the assessment of moral or ethical issues. Environment-oriented people usually feel moral obligations and are inclined to buy environment-friendly products which cause less possible harm to the environment (Saleki et al., 2019). Moral responsibility is stated as an individual norm (Manstead, 1999). Grounded on the individual responsibility and beliefs of this norm, a person will show desire and intention to do a specific act. Moral responsibility denotes whether a person perceives an obligation to do an action in a moral way (Beck and Ajzen, 1991). Past literature has studied moral responsibilities in the TPB model to forecast individuals’ intentions in a different context, such as waste sorting (Wang et al., 2021), green food purchase intention in the context of COVID-19 (Qi and Ploeger, 2021), energy-saving intention and behavior (Qalati et al., 2022), and electric vehicle intention (Shalender and Sharma, 2021). It has been stated that moral norms can enhance green purchase intention (Saleki et al., 2019; Liu et al., 2020) and actual buying behavior (Qalati et al., 2022). However, in the specific context of showing intention or actually buying organic food, the following hypotheses are presented:

*H5a:* Moral responsibility positively affects employees' intention to buy organic food.

*H5b:* Moral responsibility positively affects employees' actual buying behavior.

### Environmental concerns

Environmental concerns could influence consumers’ behaviors and attitudes concerning organic products (Lin and Chang, 2012). Consumers mainly buy environment-friendly products due to their less environmental impact (Wei et al., 2022). Scholars suggested that consumers consider the environment and animal protection when buying products (Wong et al., 2020). It has been argued that consumers usually link the influence of their buying behavior with environmental mechanisms (Yeow and Loo, 2022). In the same vein, some customers like to stay in an environmentally proven hotel in the hope that they provide high-quality service and care for the environment (Bianchi and de Man, 2021). Eco-friendly consumers believe that if they buy eco-friendly items, producers will input more resources into producing such products (Prentice et al., 2019). In return, companies develop more environmental concerns and publicize ethical behaviors to receive a good image and fulfill consumer needs (Molinillo et al., 2020). Thus, environmentally conscious employees believe their organic product buying behavior will ultimately contribute to securing environment. Essentially, when employees worry about environmental problems and procedures being high conscious about it, they will try to reduce the negative impact on the environment. Resultantly, they prefer to buy more organic food products because it puts less pressure on the environment. This study proposes the following relationship to test this mechanism:

*H6a:* Environmental concern positively affects employees' intention to buy organic food.

*H6b:* Environmental concern positively affects employees' actual buying behavior.

### Employee’s buying intention

Organic consumption in the food industry is among the key ways to gain environmental sustenance. The most common ways to minimize the environmental effects of food from the individual’s perspective are the consumption of organic food, non-consumption of air-transported food, and less meat usage (Rana and Paul, 2017). Organic or germs free food is generated using higher biodiversity, utilization of natural resources, natural methods and procedures, avoidance of genetically altered sees, and using chemicals (European-Commission, 2016). A key issue in this industry is to develop individuals’ intentions to buy organic food. Several studies in the food consumption assessments used TPB as the theoretical guidance for assessing the factors affecting the consumers’ intentions and behaviors (Ajzen, 1991). TPB assumes that behavior is decided by the intention to perform it (Raghu and Rodrigues, 2022). Intentions seize cognitive plans and motivations and immediately shape the behaviors. The intention is a product of attitude, perceived behavioral control, and subjective norms. These factors especially perceived behavioral control, form the behaviors directly and indirectly mediated by intention (Qalati et al., 2022). TPB has shown successful implementation in an array of studies, healthy food consumption (Riebl et al., 2015), green purchase behavior (Chaudhary and Bisai, 2018), energy-saving behavior (Qalati et al., 2022), and organic food (Scalco et al., 2017) and its analytical power have been shown in a variety of assessments. Therefore, this study proposes that:

*H7:* Employee's intention to buy organic food has a positive effect on Individual actual buying behavior.

Based on the direct relationships between attitude (Singh and Verma, 2017; Ajzen, 2018; Darsono et al., 2019), perceived behavioral control (Dakhan et al., 2020; Vamvaka et al., 2020), subjective norms (Bevelander et al., 2020; Salmivaara et al., 2021; Xu et al., 2022), description norms (Doran and Larsen, 2016; Khan et al., 2022), and moral responsibility with buying intention and actual behavior; and employee’s intention to buy organic food and actual buying behavior (Scalco et al., 2017), this study proposes the following mediating relationships:

*H8:* Employee's buying intention mediates the relationship between [(a) attitude; (b) subjective norms; (c) perceived behavior control; (d) descriptive norms; (e) moral responsibility; (f) environmental concerns] and individual actual buying behavior.

**Figure 1:**
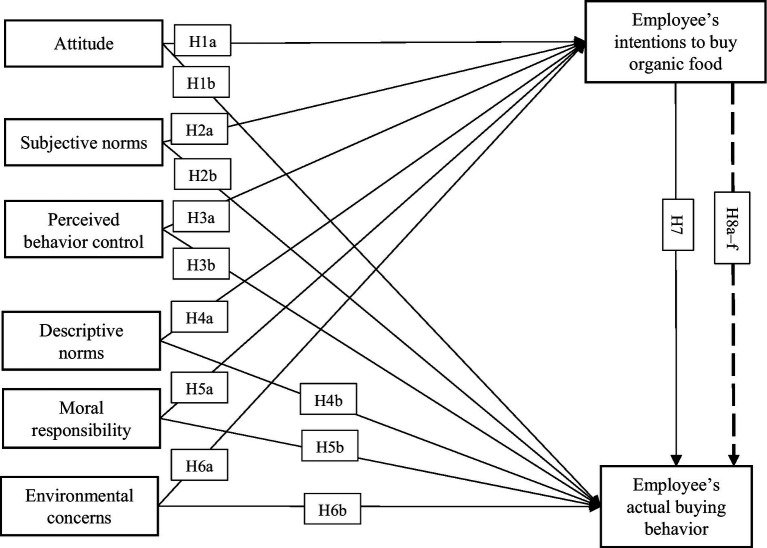
Proposed framework.

Figure 1 illustrates the proposed framework of the study and the hypothetical relationships.

## Materials and methods

### Sampling and collection of data

The study’s sample size was calculated following suggestion of 10 responses per item of the construct of Kline, (2015). This research includes 27 items of the eight constructs; hence, 270 was the required sample size. Additionally, 451 Chinese employees working in the manufacturing sector were randomly selected *via* convenient sampling. Since it is widely used in the context of buying behavior (Ahmed et al., 2021). Data were collected through a web-based survey, a widely used, cost-effective, and efficient way to reach a large audience, ensuring anonymity and recommended due to the COVID-19 disturbance (Ahmed et al., 2021; Qalati et al., 2022). The authors used cross-sectional data because it offers more opportunities for enhancing conceptual and theory development (Pesämaa et al., 2021). Additionally, it has been stated that cross-sectional analysis is adequate to provide a robust test for the existence of a correlation between constructs (Cao et al., 2007). The link was opened for 1 month from 15 June to 15 July. The administered survey comprises two portions; where first comprises the demographic variables (see Table 1).

**Table 1:** Demographical information of respondents.

Variables		Frequency	Percentage
Gender	Female	265	58.8
	Male	186	41.2
Age (years)	18–25	230	51.0
	26–35	66	14.6
	36–45	68	15.1
	Over 45	87	19.3
Education	High school/below	46	10.2
	Bachelor’s	225	49.9
	Master’s	49	10.8
	Ph.D. and other	131	29.1
Income (RMB)	Below 5,000	195	43.3
	5,001–10,000	132	29.2
	10,000–15,000	48	10.6
	Over 15,000	76	16.9
Marital status	Single	276	61.2
	Married	147	32.6
	Divorced	28	6.20

### Participants information

Table 1 reflects that 41.2% (186) were male and 58.8% (265) were female, which insights that women have more environmental concerns relative to men (Fatha and Ayoubi, 2021). Over half of them, 51.0% (230), were youngsters aged between 18 and 25. In addition, nearly half of them, 49.9% (225), had bachelor’s degrees, and 43.3% (195) had a monthly income below 5,000 RMB. Nearly two-thirds were single.

### Instruments

We have used a five-point Likert scale to measure the questions, where 1 represents strongly disagree and 5 strongly agree. The scale was adopted from the previous studies. The 10 items for the TPB factors [3 (attitude), 3 (subjective norms), and 4 (perceived behavior control)] were adopted from the previous work of (Liu et al., 2020; Sadiq et al., 2021; Lavuri, 2022). Descriptive norms (three items) were adopted from (Gao et al., 2017), and moral responsibility (four items) was adopted from (Chen, 2016). Environmental concerns measured by (four items) adopted from Le-Anh and Nguyen-To (2020) and Lavuri (2022). Employee’s buying intentions and actual buying behavior were measured by three items each. The scale from Le-Anh and Nguyen-To (2020), Liu et al. (2020), and Lavuri (2022).

## Result analysis

We used partial least square structural equation modeling (PLS-SEM) using SmartPLS 4, widely used across the disciplines (Tang et al., 2022), including consumer studies (Ahmed et al., 2021). Typically, PLS-SEM can execute a complex model comprising direct, indirect, mediating, and moderating relationships (Ostic et al., 2021; Qalati et al., 2022). To dealt with bias issues, we conducted a multicollinearity test by using the variance inflation factor (VIF) proposed by earlier scholars (Zhang and Liu, 2022). In addition, it has been criticized that Harmans’ single factor method is not an effective method to address the common method bias issues using cross-sectional data (Pesämaa et al., 2021).VIF of the latent constructs used is <5 acceptable limits (Hair et al., 2014; see Table 2). Therefore, we state that there are no multicollinearity issues. PLS-SEM analysis is a two-step approach that requires assessment of measurement and structural model (Mansoor and Wijaksana, 2022). In particular, an assessment of the measurement model is used to investigate the relationships between variables and their items, where structural model is used to explore the relationships between variables (Ru et al., 2021).

**Table 2:** Results of the measurement model.

Construct	Item code	Loading	CA	CR	AVE	Inner VIF
Attitude (Att)	Att1	0.913	0.891	0.932	0.821	1.901
	Att2	0.925				
	Att3	0.880				
Subjective norms (S.N.)	SN1	0.865	0.862	0.916	0.784	3.407
	SN2	0.917				
	SN3	0.874				
Perceived behavior control (PBC)	PBC1	0.860	0.804	0.883	0.717	3.539
	PBC2	0.842				
	PBC3	0.837				
Descriptive norms (D.N.)	DN1	0.841	0.853	0.910	0.772	2.367
	DN2	0.922				
	DN3	0.872				
Moral responsibility (M.R.)	MR1	0.881	0.900	0.931	0.771	2.142
	MR2	0.931				
	MR3	0.880				
	MR4	0.886				
Environmental concern (E.C.)	EC1	0.883	0.840	0.892	0.675	2.865
	EC2	0.839				
	EC3	0.779				
	EC4	0.780				
Buying intention (B.I.)	BI1	0.938	0.915	0.947	0.855	3.555
	BI2	0.912				
	BI3	0.925				
Actual buying behavior (ABB)	ABB1	0.935	0.926	0.943	0.871	
	ABB2	0.939				
	ABB3	0.927				

### Measurement model analysis

To conduct the reliability and validity of the constructed model, we used confirmatory factor analysis. Table 2 illustrates the reliability and validity results. In particular, Cronbach’s alpha (traditional) and composite (broadly applied) indicators for reliability. The value for both reliability indicators retained ≥0.7 benchmarks. Hence, this study met the necessary reliability (Hair et al., 2019). In addition, we tested convergent and discriminant validity. In particular, convergent validity refers to the degree to which parameters of a variable correlate with “one another.” We employed factor loading and the average variance extracted (AVE) to assess the convergent validity. The value of factor loadings retained between >0.7 and < 0.95 benchmarks (Hair et al., 2019), and AVE retained ≥0.5 benchmarks (Fornell and Larcker, 1981; Fan et al., 2021); thus, we conclude a strong convergence of the parameters used in the particular variables.

Discriminant validity refers to the extent to which employed variables must be uncorrelated (Fornell and Larcker, 1981). It has been stated that if the square root of the AVE of the construct is higher than the correlations among the variables, it is satisfactory (Fornell and Larcker, 1981; see Table 3). Additionally, heterotrait-monotrait ratio (HTMT) criteria have been employed to conduct the discriminant validity test proposed by (Henseler et al., 2015). The recommended value should be <0.90. Table 3 reflects that all values are retained below 0.90, and the majority of them are less than the 0.85 benchmarks (Hair et al., 2019). Thus, based on the results, we conclude that the measurement model has adequate reliability and validity.

**Table 3:** Discriminant validity.

Constructs	ABB	Att	BI	DN	E.C.	MR	PBC	SN
**Fornell–Larker criterion**								
Actual buying behavior (ABB)	**0.933**							
Attitude (Att)	0.333	**0.906**						
Buying intention (B.I.)	0.785	0.471	**0.925**					
Descriptive norms (D.N.)	0.694	0.356	0.629	**0.879**				
Environmental concern (E.C.)	0.628	0.560	0.742	0.522	**0.822**			
Moral responsibility (MR)	0.625	0.394	0.672	0.518	0.655	**0.878**		
Perceived behavior control (PBC)	0.706	0.586	0.745	0.680	0.693	0.594	**0.846**	
Subjective norms (S.N.)	0.663	0.608	0.705	0.690	0.613	0.469	0.753	**0.886**
**Heterotrait-monotrait (HTMT) ratio**								
Actual buying behavior (ABB)								
Attitude (Att)	0.366							
Buying intention (B.I.)	0.852	0.520						
Descriptive norms (D.N.)	0.772	0.404	0.702					
Environmental concern (E.C.)	0.688	0.657	0.832	0.588				
Moral responsibility (MR)	0.678	0.441	0.736	0.579	0.746			
Perceived behavior control (PBC)	0.812	0.686	0.855	0.811	0.823	0.695		
Subjective norms (S.N.)	0.740	0.695	0.792	0.801	0.710	0.529	0.893	

Bold values used to show correlations.

### Structural model analysis

After the assessment of the measurement model, in the next step, we assessed the inner model through the coefficient of determination (*R*^2^), hypotheses testing, and effect size (*f*^2^). Regarding the hypotheses testing, we utilized the bootstrapping method with 5,000 subsamples for 451 cases to produce path results at *p* = 0.05. Table 4 illustrates that except (*H4a*, *H6b*, and *H8d*), all hypotheses were supported. Among the direct hypotheses, environmental concern strongly influences (β = 0.302) buying intention toward organic food, whereas on actual buying behavior, buying intention has a strong influence (β = 0.405). Related to the mediation, Baron and Kenny (1986) stated if a direct relationship fails to be significant, a significant indirect relationship indicates full mediation; however, if both direct and indirect relations present significant effects, partial mediation is indicated. In this way, we conclude that for *H8e,* buying intention fully mediates the relationship between environmental concerns and actual buying behavior.

**Table 4:** Results of the structural model, common method bias, and model fit.

Hypothesis	Relationship	β	S.D.	*t*-value	Supported	*f* ^2^
*Direct effect*						
*H1a*	Att → BI	−0.101^**^	0.037	2.277	Yes	0.020
*H1b*	Att → ABB	−0.183^**^	0.039	4.650	Yes	0.061
*H2a*	SN → BI	0.276^***^	0.05	5.564	Yes	0.019
*H2b*	SN → ABB	0.137^**^	0.056	2.454	Yes	0.086
*H3a*	PBC → BI	0.217^***^	0.048	4.503	Yes	0.050
*H3b*	PBC → ABB	0.157^**^	0.051	3.067	Yes	0.024
*H4a*	DN → BI	0.051	0.037	1.383	No	0.004
*H4b*	DN → ABB	0.214^***^	0.044	4.860	Yes	0.068
*H5a*	MR → BI	0.229^***^	0.042	5.393	Yes	0.095
*H5b*	MR → ABB	0.130^**^	0.038	3.458	Yes	0.027
*H6a*	EC → BI	0.302^***^	0.043	7.035	Yes	0.128
*H6b*	EC → ABB	0.040	0.049	0.808	No	0.002
*H7*	BI→ABB	0.405^***^	0.056	7.240	Yes	0.161
*Indirect effect*					*Mediation level*	
*H8a–f*	Att → BI→ABB	−0.041^**^	0.016	2.539	Yes	Partial
	SN → BI→ABB	0.112^***^	0.026	4.292	Yes	Partial
	PBC → BI→ABB	0.088^***^	0.022	3.973	Yes	Partial
	DN → BI→ABB	0.021	0.015	1.342	No	No
	MR → BI→ABB	0.093^***^	0.020	4.553	Yes	Partial
	EC → BI→ABB	0.122^***^	0.026	4.648	Yes	Full

Critical values. TPB model: *R*^2^ (BI) = 0.605 and *R*^2^ (ABB) = 0.679. Extended TPB model: *R*^2^ (BI) = 0.719; *R*^2^ (ABB) = 0.714. Goodness of fit indices: SRMR = 0.065

** *p* < 0.01;

*** *p* < 0.001.

*R*^2^ is another measure used to explain the variations and show the nomological and predictive validity and the explanatory power of the structured model on a scale of 0–1 (Sultan et al., 2020). *R*^2^ value of >0.75, 0.5, and ≤ 0.25 is considered strong, moderate, and weak effect, respectively (Hair et al., 2019). Table 4 illustrates that buying intention explains 71.9% of the variance of the TPB factors and the extended factors and actual buying behavior explains 71.4% of the TPB, buying intention, and extended factors.

Table 4 also reflects the *f*^2^ (effect size) of the variables. The effect size value is utilized to assess the relevance of variables in explaining selected endogenous variables (Hair et al., 2019). *f*^2^ value of 0.35, 0.15, and 0.02 is considered large, medium, and small effect (Le-Anh and Nguyen-To, 2020). Table 4 shows that subjective and descriptive norms have a small effect on buying intention and environmental concern has a small effect on actual buying behavior. At the same time, the levels of other interactions in the proposed model are at medium to a large impact. Finally, regarding the model fit, we used the standardized root mean square residual (SRMR) proposed by (Hair et al., 2019). Table 4 illustrates that the SRMR value is 0.065, which is far below the 0.08 benchmark (Hair et al., 2019). Hence, we conclude that the present research structural model has an adequate, satisfactory level.

## Discussion

Regarding the effect of attitude, the above findings evidenced a negative and significant impact of attitude on Chinese individual intention (*β* = −0.101, *p* = 0.006) and actual buying behavior (*β* = −0.183, *p* = 0.000); thus, *H1(a–b)* were supported. People hold a negative attitude toward organic food because they think that organic products are expensive and negatively impact their income. This study result is consistent with those (Zboraj, 2021), who reported that organic food prices have risen by 13.9% in 2019 and are constantly increasing because of higher demand and production costs. These findings supported the previous work of (Suh et al., 2012). Regarding the second factor of the TPB, we found a positive and substantial effect of subjective norms on an individual’s buying intention (*β* = 0.276, *p* = 0.000) and actual buying behavior (*β* = 0.137, *p* = 0.014); thus, *H2(a–b)* were supported. This finding infers that families, friends, and those we care for play a critical role in the organic food buying intention and actual buying behavior. This result of the study is in line with previous work of (Testa et al., 2019; Ahmed et al., 2021; Sadiq et al., 2021), who highlighted that subjective norms strongly affect organic food buying intention and actual buying behavior.

In addition, the third factor (perceived behavior control) of the TPB theory was also found with a positive and significant effect on an individual’s buying intention (*β* = 0.217, *p* = 0.000) and actual buying behavior (*β* = 0.157, *p* = 0.002); thus, *H3(a–b)* were supported. This outcome implies that individuals have complete control over whether or not to buy organic products, whether or not to eat, and have the ability and enough resources to buy organic food. This result is consistent with previous work of (Sultan et al., 2020; Sadiq et al., 2021).

Regarding the first extended factor (descriptive norms), we could not find support for the effect of descriptive norms on buying intention (*β* = 0.051, *p* = 0.167 > 0.05); thus, *H4(a)* was not supported. While we found its significant effect on actual buying behavior (*β* = 0.214, *p* = 0.000); thus, *H4(b)* was supported. This finding infers that descriptive norms have the second strongest influence on actual buying behavior. It furthers that when family, friends, and to whom we care to participate in buying organic food, individuals are more likely to do things and replicate the behavior. This finding is consistent with the work of Ham et al. (2015) and Qalati et al. (2022) who identified the importance of descriptive norms and separated it from the subjective norms in the context of organic food and energy-saving behavior.

Regarding the second extended factor (moral responsibility), we found a significant effect of moral responsibility on an individual’s buying intention (*β* = 0.229, *p* = 0.000) and actual buying behavior (*β* = 0.130, *p* = 0.001); thus, *H5(a–b)* were supported. This result states that a 1 unit change in this factor led to a 22.9 and 13% change in buying intention and buying behavior, respectively. It furthers that people feel guilty about not buying, realize their obligation to protect the environment, and perform organic food consumption. These outcomes are consistent with studies of (Liu et al., 2020; Wang et al., 2021).

Finally, regarding the final extended factor (environmental concern), we found its significant effect of environmental concern on buying intention (*β* = 0.302, *p* = 0.000); thus, *H6(a)* was supported. In contrast, we could not find support for its effect on actual buying behavior (*β* = 0.040, *p* = 0.419 > 0.05); thus, *H6(b)* was not supported. These results are consistent with those (Maichum et al., 2016; Ahmed et al., 2021; Ru et al., 2021; Shalender and Sharma, 2021).

In addition, this research evidenced a positive and significant effect of buying intention on actual buying behavior toward organic food in the context of the Chinese market (*β* = 0.405, *p* = 0.000); thus, *H7* was supported. This finding indicated that buying intention has the strongest influence on buying behavior. It implies that 1 unit change in intention led to a 40.5% change in actual buying behavior. This result is supported by previous work of (Chaudhary and Bisai, 2018; Sultan et al., 2020; Lavuri, 2022).

Furthermore, this research evidenced the mediating role of individual intention to buy organic food between the TPB and two extended factors (moral responsibility and environmental concerns; *p* < 0.01 and 0.001); thus, except *H8c*, all mediation hypotheses *H8(a, b, d, e, and f)* were supported. This result is consistent with prior work by Sultan et al. (2020), which reported the mediation of intention between the TPB factors and behavior in Australia’s context of organic food consumption. Typically, buying intention partially mediated the relationship between attitude, subjective norms, perceived behavior control, moral responsibility, and actual buying behavior, while fully mediated between environmental concern and actual buying behavior. The proposed extended model explains 71.9% (buying intention) and 71.4% (buying behavior). Moreover, in terms of the explanatory power difference between the original TPB (60.5% for buying intention) and the extended model (71.9% for buying intention) is >11.4%. This result is in line with recent findings of Chen (2016) and Qalati et al. (2022), who proposed that including descriptive norms and moral obligation increases individual intention.

### Theoretical contribution

Our research findings have offered several theoretical contributions. First, several scholars have explored the TPB factors to predict green consumption intention (Sultan et al., 2020; Ahmed et al., 2021; Sadiq et al., 2021). However, few studies extended the TPB framework in the context of organic food consumption, such as (Le and Nguyen, 2022). Most of them extended the TPB theory to predict intention (Ahmed et al., 2021; Lavuri, 2022; Le and Nguyen, 2022), while rarely studies (Testa et al., 2019) explored both intentions and actual buying behavior. In this context, this is the first study that extended the TPB model in the context of individuals’ intentions and buying behavior by descriptive norms, moral responsibility, and environmental concerns.

Second, it has been called for upcoming studies in developing countries regarding how individuals act against increasing demand for green consumption (Rana and Paul, 2017). In this context, we filled this gap by improving our understanding of the determinants influencing green purchase intention and buying behavior in one of the leading emerging markets and a country responsible for the export of organic products. Our results illustrate that out of the six factors; attitude negatively influences intention and actual buying; this could be because of higher prices to buy organic food (Wei et al., 2022). While subjective norms strongly influence consumption intention and buying intention, followed by environmental concern having a strong impact on actual buying behavior.

Finally, many scholars have explored the TPB’s and extended factors’ influences on an individual’s intention (Maichum et al., 2016; Liu et al., 2020; Le and Nguyen, 2022) and actual buying behavior (Testa et al., 2019). A few studies examined the mediating role of individual intention to buy green food between the TPB and extended factors. Such as Sultan et al. (2020) investigated the mediation of consumption intention between the TPB factors and actual buying behavior. Likewise, Qalati et al. (2022) investigated the context of energy-saving behavior and called for future studies to examine its effect between the TPB and extended factors. Therefore, this study filled this research gap by investigating the mediation of intention between the TPB and extended factors. Our result evidenced that buying intention significantly mediated the relationship between five factors except for descriptive norms.

### Practical implications

This research also offers several implications for practitioners. First, individuals’ negative attitude insights the organic food providers and governments to seek ways to produce the product at less cost so that supply meets the demand (Zboraj, 2021). In addition, the government must provide subsidies to farmers to make the product available on time and advertise the benefits and consequences of using non-organic foods. In this way, people are aware of their interest and responsibility for buying organic food.

Second, a positive effect of factors (subjective norms, perceived behavior control, moral responsibility, and environmental concern) on intentions. And a significant effect of (subjective norms, perceived behavior control, descriptive norms, moral responsibility, and environmental concern) on actual buying behavior suggests that practitioners develop strategies for public advertisement, launch a general message and campaigns both in urban and rural areas to prevent the environmental sustainability, and increase the awareness related to organic consumptions. Consequently, the increased demand motivates the farmers to increase production, which at large helps the government reduce the inequality gap between the poor and rich.

Lastly, a significant effect of descriptive norms on buying behavior illustrates that role model among family member, friends, and others (important to us) must perform the role of ambassadors and improves the act of buying organic consumption. In addition, the significant effect of moral responsibility and environmental concerns on intention suggests that practitioners of environment focus companies must include the content associated with environmental sustainability and moral responsibility in their promotional campaigns as it may lead to actual buying behavior.

### Limitations and future research

This study is not free from the limitations that offer future scholars pathways. First, our study explored the antecedents and consequences of the TPB factors and three extended factors, namely descriptive norms, moral responsibility, and environmental concerns on individuals buying intention and actual buying behavior in the context of China, which could be one of the limitations. Organic food consumption is a global concern; therefore, we suggest that future studies can replicate the model in different regions and countries to validate the results. Second, data collection *via* a web-based survey could be another limitation because it enabled those with internet access and deprived those without access. Therefore, we suggest future studies in the market or in a real-time survey (where people buy organic or non-organic food) to better validate the results (especially the result of the attitude, descriptive norms, and environmental concerns). Third, our study separately used subjective and descriptive norms to understand their individual impacts better. However, both norms are sub-part of social norms; this can be a third limitation. Therefore, we suggest that future scholars use social norms as a single factor by combining both factor parameters. Fourth, this research explores the effect of six factors on buying intention. However, there could be several other factors, such as environmental awareness, knowledge and consciousness, price, health consciousness, food quality, certification, social-cultural factors, etc.

## Conclusion

Globally, the manufacturing sector is criticized for increasing environmental degradation and pollution. In this context, this research was conducted in the manufacturing sector and had twofold objectives (to explore the antecedent and consequences of the employee intention toward organic food consumption and investigate the mediating role of employee buying intention). Using a convenient sample, 451 Chinese employees were selected, and analysis was conducted through PLS–SEM techniques using SmartPLS 4. The findings express that employees hold a negative attitude toward buying organic food because it is expensive. In addition, subjective norms and perceived behavior control have a positive and significant impact on employee intention and actual behavior. Among the extended factors, the descriptive norm has a positive effect on employees’ actual buying behavior. In addition, moral responsibility has a significant influence on both buying intention and actual behavior. In contrast, environmental concerns only have a significant influence on buying intention. Lastly, employee buying intention partially mediated the link between attitude, subjective norms, perceived behavior control, moral responsibility, and actual buying behavior, while fully mediated between environmental concern and actual buying behavior.

## Data availability statement

The raw data supporting the conclusions of this article will be made available by the authors, without undue reservation.

## Ethics statement

The studies involving human participants were reviewed and approved by Ethics committee of Wuhan Donghu University, China. The patients/participants provided their written informed consent to participate in this study.

## Author contributions

All authors listed have made a substantial, direct, and intellectual contribution to the work and approved it for publication.

## Conflict of interest

The authors declare that the research was conducted in the absence of any commercial or financial relationships that could be construed as a potential conflict of interest.

## Publisher’s note

All claims expressed in this article are solely those of the authors and do not necessarily represent those of their affiliated organizations, or those of the publisher, the editors and the reviewers. Any product that may be evaluated in this article, or claim that may be made by its manufacturer, is not guaranteed or endorsed by the publisher.

## Figures and Tables

**Table:** 

Attitude	“It would be beneficial to use organic product”
	“I would have positive feelings toward organic products”
	“The organic product is safe and healthier for everyone”
Subjective norms	“My family thinks that it is a good idea to buy and use organic products”
	“People whose opinions I value would prefer organic products”
	“The good opinion of my friends motivates me to buy organic products”
Perceived behavior control	“I have complete control over whether or not to consume organic foods regularly instead of conventional foods”
	“Whether or not I eat organic foods is entirely up to me”
	“I have enough resources to consume organic foods”
Descriptive norms	“My parents have acted to buy organic products in our family”
	“My family has participated in organic product consumption behavior”
	“Others who are close to me have participated in organic product consumption behavior”
Moral responsibility	“I would feel guilty about not buying eco-friendly /organic products”
	“It is my moral responsibility to buy organic products to protect the environment”
	“It is my moral responsibility to stop buying not organic products”
	“It is my moral responsibility to perform organic product consumption behavior”
Environmental concern	“Organic products help to sustain the natural environment”
	“Organic products minimize waste and recycling”
	“I have avoided buying normal products because they had potentially harmful environmental effects”
	“I am very concerned about the state of the natural environment”
Buying intention	“I intend to buy organic products rather than normal products”
	“I prefer to spend more than average on eco-friendly products”
	“I shall consider changing to eco-friendly brands with respect to ecological issues”
Actual buying behavior	“I often purchased organic products”
	“I always prefer organic products even though the prices are high”
	“I often recommend others to consume organic products”
